# Effectiveness and Clinical Performance of Erythritol Air-Polishing in Non-Surgical Periodontal Therapy: A Systematic Review of Randomized Clinical Trials

**DOI:** 10.3390/medicina58070866

**Published:** 2022-06-28

**Authors:** Florin Onisor, Alexandru Mester, Leonardo Mancini, Andrada Voina-Tonea

**Affiliations:** 1 Department of Maxillofacial Surgery and Implantology, University of Medicine and Pharmacy “Iuliu Hatieganu”, 400012 Cluj-Napoca, Romania; florin.onisor@umfcluj.ro; 2 Department of Oral Health, University of Medicine and Pharmacy “Iuliu Hatieganu”, 400006 Cluj-Napoca, Romania; 3 Department of Life, Health and Environmental Sciences, University of L’Aquila, 67100 L’Aquila, Italy; leonardo.mancini@graduate.univaq.it; 4 Department of Dental Materials, University of Medicine and Pharmacy “Iuliu Hatieganu”, 400012 Cluj-Napoca, Romania; andrada.tonea@umfcluj.ro

**Keywords:** erythritol, air-polishing, periodontal disease, periodontitis, non-surgical periodontal therapy

## Abstract

*Background and objectives*: The purpose of the present systematic review was to analyze the effectiveness of erythritol-based air-polishing in non-surgical periodontal therapy. *Materials and methods*: The protocol details were registered in the PROSPERO database (CRD42021267261). This review was conducted under the PRISMA guidelines. The electronic search was performed in *PubMed, Scopus*, and *Web of Science* databases to find relevant clinical trials published until January 2022. The inclusion criteria consisted of human clinical trials which reported the use of non-surgical periodontal treatment and erythritol air-polishing compared to non-surgical periodontal treatment alone in patients with good systemic health requiring treatment for periodontal disease. *Results*: 810 studies were imported into the Covidence Platform. Of these, seven clinical trials met the inclusion criteria. In active periodontal therapy, for PD (probing depth), CAL (clinical attachment level), and BOP (bleeding on probing), no statistical significance was achieved at 6 months follow-up. In supportive periodontal therapy for PD, CAL, and BOP, no statistical significance was achieved at 3 months follow-up. *Conclusions*: The findings suggest that erythritol air-polishing powder did not determine superior improvements of periodontal parameters compared to other non-surgical periodontal therapies. Future randomized clinical trials (RCTs) with calibrated protocols for diagnosis, therapeutic approaches, and longer follow-up are needed to draw a clear conclusion about the efficiency of erythritol air-polishing powder.

## 1. Introduction

Periodontal disease represents a public health issue and occupies the sixth place worldwide among the most common oral pathologies, with a prevalence of 11.2% [1]. It is well-known that periodontal disease is determined by the accumulation of microbial biofilms, which will initiate the formation of periodontal pockets and clinical attachment loss [1]. The first therapy that is indicated in a periodontitis patient is the non-surgical periodontal therapy [2]. The purpose of the non-surgical therapy is to provide the elimination of radicular microorganisms and endotoxins by eliminating the supra- and subgingival plaque deposits in order to obtain periodontal healing [2]. 

Active periodontal therapy (APT) represents the totality of conventional treatment approaches, which includes oral care recommendations and eradication of bacteria, accompanied by additional antimicrobial treatment [1,2,3]. Together with the elimination of risk factors, ultrasonic and manual scaling procedures play an essential role in the context of APT, being responsible for achieving stability of the periodontal status [1,2,3]. APT aims to set the parameters for preventing periodontal deterioration and minimization of deep periodontal pockets and implicitly for tooth and periodontal attachment preservation [3]. The boundaries of APT are defined by the nonappearance of pocket defects deeper than 4 mm, accompanied by bleeding on probing (BOP) or by the nonappearance of probing depths (PD) equal or more than 6 mm [4].

When endpoints of APT have been reached, patients must undergo a maintenance phase known as supportive periodontal therapy (SPT) in order to preserve the results obtained during the active periodontal therapy [4]. SPT is focused on the minimization of reinfection episodes and on preventing the evolution of the pathology, by sustaining patient comfort, without the occurrence of acute symptoms or increased tooth mobility. The main goal of SPT is to inhibit newly generated supra- and subgingival microbiota as well as recent dental deposits [4]. Manresa and coworkers underline that SPT should cover all aspects of a standard dental assessment, which includes periodontal reexamination with risk analysis, removal of dental plaque, and if necessary, calculus supra and subgingival reinterventions on sites with unremitting disease [5]. 

Thus far, air-polishing powders are used in the supra- and subgingival periodontal procedures. During the treatment of remnant periodontal pockets or during SPT, air-polishing powders can be used as a substitute for manual and ultrasonic procedures in order to minimize inflammatory episodes and to remove microbial load [6]. The negative impact of abrasive sodium bicarbonate on the tooth surface determined the introduction of other components, such as erythritol- or glycine-based powder [7]. Sodium bicarbonate air-polishing powders were proven to be more harmful than other powder-based products, generating soft and hard tissue alterations involving volume and depth modifications [7]. Furthermore, sodium bicarbonate powder was shown to have more negative effects on the periodontal cell density and viability when compared to other similar products [8]. Recently, erythritol powder air-polishing (EPAP) has been introduced in APT and SPT [4]. The use of this product indicated non-traumatic effects onto periodontal tissues and improved periodontal parameters; some limitations have been reported, such as limitations in removing large deposits of calculus or other deposits [4].

Therefore, the purpose of this systematic review with meta-analysis was to evaluate the effectiveness of erythritol-based air-polishing in non-surgical periodontal therapy.

## 2. Materials and Methods

### 2.1. Protocol Registration

A priori, the protocol of this review was registered into the PROSPERO database (CRD42021267261). 

### 2.2. Eligibility Criteria

The research question of the present study was: “What is the effectiveness of using erythritol air-polishing as an adjunct in non-surgical periodontal therapy?” Inclusion criteria according to PICOS (Participants, Interventions, Comparison, Outcome, Study design) criteria were:

Participants: Patients in good systemic health requiring treatment for periodontal disease;

Interventions: Non-surgical periodontal treatment and erythritol air-polishing;

Comparison: Non-surgical periodontal treatment without erythritol air-polishing; 

Outcome: Changes in periodontal parameters recorded: probing depth (PD), clinical attachment loss (CAL), and bleeding on probing (BOP);

Study design: randomized clinical trials (RCTs), either of a split-mouth design or a parallel-group, clinical trials.

The following exclusion criteria were applied: in vitro, animal studies; cross-sectional, cohort studies; systematic or narrative reviews, case reports, case series, monographs, or letters to the editor; missing data regarding periodontal parameters or no reports on the use of erythritol; insufficient/missing/unpublished data; or articles published in other languages than English.

### 2.3. Search Process

The electronic search was performed by two independent reviewers (F.O. and A.M.) on *PubMed, Scopus*, and *Web of Science* databases until January 2022. To identify relevant articles, the following search strategy was applied: (“erythritol” OR “erythritol powder” OR “erythritol air polishing powder”) AND (“nonsurgical periodontal therapy” OR “non-surgical periodontal therapy” OR “periodontal therapy” OR “scaling and root planning” OR “nonsurgical periodontal treatment” OR “non-surgical periodontal treatment” OR “periodontal treatment” OR “periodontitis” OR “periodontal disease” OR “gingivitis” OR “gingival disease”). Titles and abstracts were screened for eligibility, and irrelevant articles were excluded. In the second phase, after removing the duplicates, full-text articles previously obtained were examined, and those that corresponded to the inclusion criteria were downloaded. In case of any disagreements, a third reviewer (A.V-T.) intervened with an additional discussion. 

### 2.4. Data Extraction

The following data from the included studies were taken: first author, year of study, country, reference, RCT type, characteristics of participants, type of intervention, periodontal parameters, follow-up, outcomes, and conclusions. 

### 2.5. Quality Assessment

The risk of bias (RoB) was quantified using Cochrane RoB, version 2.0 [9]. For each clinical trial included in the analysis, a number of seven domains were assessed as follows: random sequence generation; allocation concealment; blinding of participants and/or personnel involved in the study; blinding of assessors; incomplete outcome data reporting; selective reporting of outcomes; and other sources of bias. These domains received the quality of low, unclear, or high. RoB criteria were assessed by two independent reviewers (A.M., L.M.); if any disagreement was present, a third reviewer (F.O.) intervened. 

### 2.6. Data Synthesis

In case of a consistent number of RCTs reporting the same parameters, a meta-analysis was performed using Review Manager (RevMan) [Computer program] (Version 5.4.1, The Cochrane Collaboration, 2020) [10]. A random effect model with a confidence interval (CI) of 95% was used. The heterogeneity among the studies was evaluated with a I-squared statistic test (I^2^). I^2^ values lower than 30% indicated low heterogeneity, values between 30–60% indicated moderate heterogeneity, and values over 60% indicated substantial heterogeneity. 

## 3. Results

### 3.1. Study Selection

**Figure 1:**
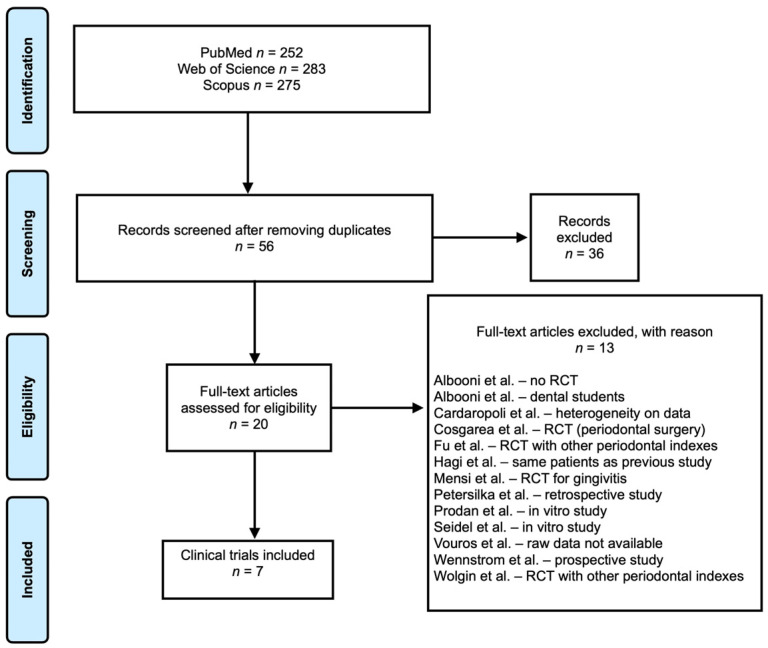
Prisma flowchart.

A total of 810 studies were retrieved from *PubMed, Web of Science*, and *Scopus* databases. After removing the duplicates, a total of 56 studies were assessed through title and abstract. From these, 20 articles were full-text assessed. In the end, 7 clinical trials met the inclusion criteria. The flow diagram according to PRISMA guidelines is shown in Figure 1. 

### 3.2. Study Characteristics

#### 3.2.1. Description of the Included Studies

The studies were published between 2013 and 2021 and were conducted in Korea, Germany, Italy, Switzerland, and Norway. In regards to the study design, four RCTs (three parallel RCTs and one split-mouth RCT) focused on active periodontal therapy, and three RCTs (two parallel RCTs and one split-mouth RCT) focused on supportive periodontal therapy (Table 1).

#### 3.2.2. Characteristics of Participants 

The cohort included varied between 20 and 180 participants (Table 1). Mean age varied between 48.44 ± 9.31 and 61 years. The gender cohort varied, in females, between 7 and 29 and in males, between 6 and 23. Only one RCT did not report the mean age and the gender. Periodontal indexes assessed were probing depth (PD), clinical attachment level (CAL), and bleeding on probing (BOP). Diagnosis of the participants was moderate to advanced chronic periodontitis or periodontitis stage III–IV. The control group was treated using SRP (scaling and root planning) alone, SRP and supragingival erythritol powder air-polishing, quadrant-wise SRP (Q-SRP), full-mouth scaling (FMS), or full-mouth disinfection (FMD). The test group was treated using SRP+ EPAP (erythritol powder air-polishing powder), SRP + supragingival and subgingival EPAP, FMD with adjuvant erythritol air-polishing, or subgingival erythritol air-polishing. Follow-up varied from 1 to 12 months. 

### 3.3. Risk of Bias Assessment

**Figure 2:**
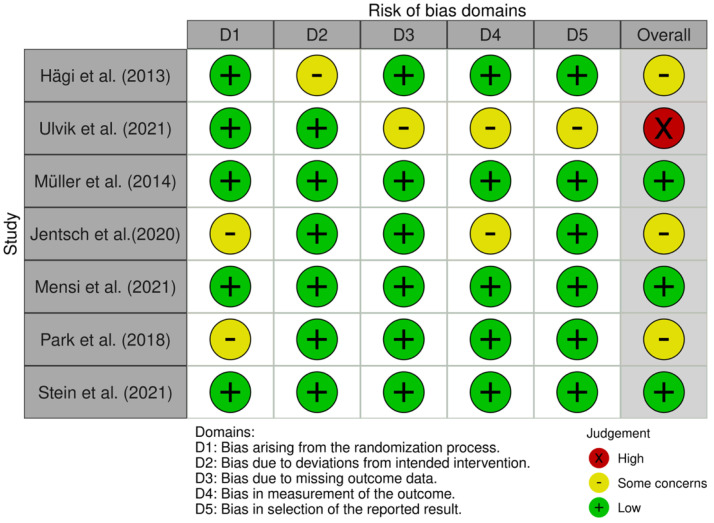
Cochrane RoB quantification [11,12,13,14,15,16,17].

Among the seven RCTs included, three RCTs were included as low-risk, three RCTs were included as some concerns, and one RCT had high risk (Figure 2).

### 3.4. Quantitative Data Analysis

**Figure 3:**
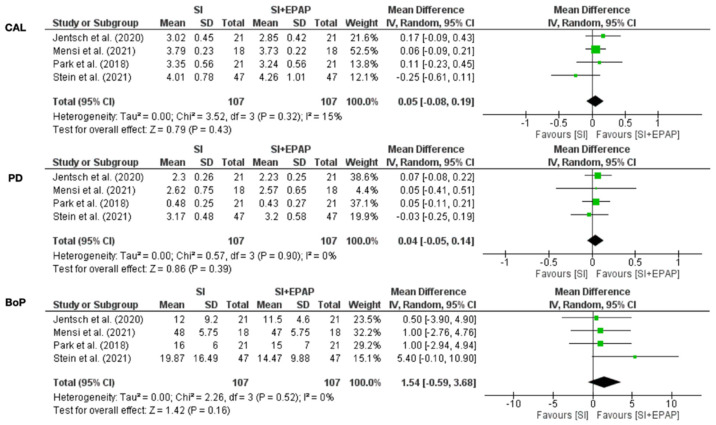
Erythritol in active periodontal therapy.

**Figure 4:**
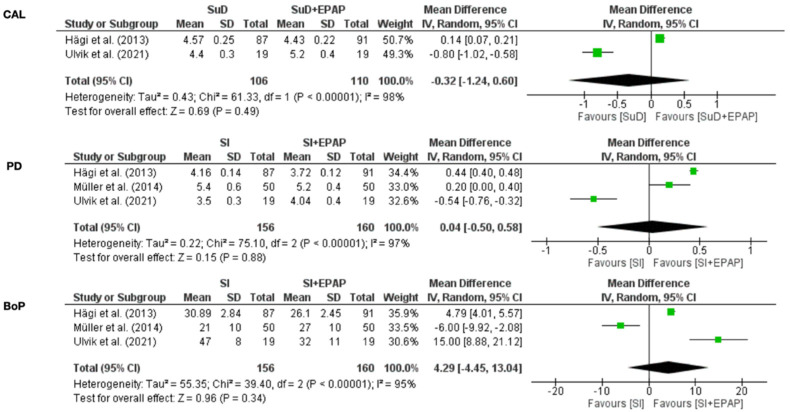
Erythritol in supportive periodontal therapy.

In active periodontal therapy, 6 months follow-up was considered. For periodontal parameters PD, CAL, and BOP, no statistical significance was achieved although I^2^ values for all three parameters indicated low heterogeneity (Figure 3). In supportive periodontal therapy, 3 months follow-up was considered. For periodontal parameters PD, CAL, and BOP, no statistical significance was achieved; I^2^ values for all three parameters indicated substantial heterogeneity (Figure 4).

## 4. Discussion

Our systematic review with meta-analysis evaluated the efficiency of erythritol air-polishing in non-surgical periodontal therapy (APT and SPT). Our findings suggest no superior efficiency in using erythritol air-polishing. In APT, forest plots graphics for periodontal parameters (PD, CAL, BOP) did not achieve statistical significance. An addition, in SPT, forest plot graphics for the same periodontal parameters did not achieve statistical significance. 

What can be depicted is that EPAP may be efficiently used in combination with hand or ultra-sonic scaling and root planning in order to control the levels of biofilm and reduce the periodontal inflammation. Although erythritol powder is used as an adjuvant to non-surgical periodontal therapy, it does not greatly reduce periodontal parameters in comparison to SRP procedures. In the meta-analysis of Abdulbaqi and coworkers [4], statistical significance was obtained for EPAP during APT for CAL parameter (0.16 mm; *p* < 0.02). The authors concluded that EPAP may be used as adjunct in APT and as an alternative to mechanical debridement in SPT. Furthermore, in the systematic review of Nascimento and coworkers [6], air-polishing showed no superior difference in comparison to SRP. Furthermore, a recent RCT published by Divnic-Resnik and coworkers concluded that EPAP could be efficient in reducing initial periodontal pockets with PD ≥ 5.5 mm [18]. On the other hand, the systematic review from Zhang and coworkers [19] concluded no efficiency of subgingival air-polishing compared to subgingival ultrasonic debridement during SPT.

Advantages, disadvantages, and side effects of different air-polishing powders may represent a challenge for choosing the suitable option for APT and SPT. Although erythritol, glycine, or sodium bicarbonate powders showed similar effects to standard approaches [20], glycine and erythritol powders were considered to be recommended due to the fact that sodium bicarbonate enhanced abrasiveness [21]. Moreover, Sahrmann and coauthors underlined that the use of sodium bicarbonate powder in patients with visible radicular surfaces should be contraindicated in patients; instead, less-coarse powders should be used [22]. A study conducted by Tsang and coworkers reported less soft tissue damage when using glycine powders compared to sodium bicarbonate powder in air-polishing devices [23]. Nevertheless, the study of Bosland et al. concluded that glycine and erythritol are recommended for subgingival instrumentation due to their low-abrasion capacity, while powders with bigger particles (such as sodium bicarbonate) are indicated in supragingival approaches [24,25,26]. On the other hand, the research from Kroger and coworkers showed that at the level of dentin, erythritol produces limited hard tissues damage [27,28]; the low abrasiveness and the antimicrobial capacity recommends erythritol as the product of choice in removing subgingival biofilm. 

The research from Hashino and coworkers confirmed the capacity of erythritol to inhibit *Porphyromonas gingivalis* and *Streptococcus gordonii*, two known components of the subgingival biofilm [25]. Furthermore, when compared to the conventional glycine powder, the study published by Drago proved that erythritol–chlorhexidine powder is superior in the process of inhibiting bacteria, such as *Bacteroides fragilis* and *Staphylococcus aureus*, and even yeasts *Candida albicans* [26]. 

Consequences of air-polishing powders should be regarded when deciding the appropriate treatment decision. A scanning electron microscopy study from Herr and coworkers demonstrated that sodium bicarbonate powder produces an extended exposure of the dentinal tubules when compared to other low-abrasive powders based on glycine [29]. Not only the effect on the hard dental tissue but also the gingival deterioration must be taken into consideration. Low-abrasive air-polishing products based on glycine formulas were proven to produce less erosions of the soft tissue compared to sodium bicarbonate or even manual scaling procedures [28]. Erythritol was as well-recognized to cause less soft tissue damage, allowing a greater acceptability on the patient’s behalf [20]. 

One of the difficulties encountered in this systematic review was the differences of non-surgical periodontal therapy protocols used. For example, in APT, for the test group, Park et al. [11] used EPAP and SRP; Jentsch et al. [12] used subgingival instrumentation + subgingival EPAP; Mensi et al. [13] used ultrasonic instrumentation and supragingival and subgingival EPAP; and Stein et al. [14] used FMD and EPAP. In SPT, same differences were encountered: Hagi et al. [15] used EPAP, while Muller et al. [16] and Ulvik et al. [17] used subgingival EPAP. Therefore, utilizing different protocols the results represented a bias, and accurate statistical analysis could not be achieved. Another limitation consisted of different methods to assess the diagnosis of periodontitis, to evaluate the periodontal parameters, and to establish the follow-up after periodontal therapy. Although the literature search was limited to a few databases and was done until January 2022, several data sources may have been left out from the analysis.

## 5. Conclusions

The findings of the present systematic review indicated that erythritol air-polishing powder did not determine superior improvements of periodontal parameters compared to other non-surgical periodontal therapies in systemically healthy patients diagnosed with periodontitis. In addition, erythritol air-polishing powder can be successfully used in non-surgical periodontal therapy for biofilm control. Future RCTs with longer follow-up periods and standardized protocol for diagnosis and therapy are needed to draw a clear conclusion about the efficiency of erythritol air-polishing powder.

## Figures and Tables

**Table 1:** Characteristics of the included studies.

Author. Year. Country	Study Type	Participant’s Characteristics	Type of Intervention	Periodontal Parameters	Follow-Up	Outcomes	Conclusion
Active periodontal therapy							
Park, 2018, Korea [11]	RCT Split mouth	*n* = 21 Control group Mean age: NA Female: *n* = NA Male: *n* = NA Test group Mean age: NA Female: *n* = NA Male: *n* = NA Diagnosis: moderate chronic periodontitis	Control: SRP Test: SRP+ EPAP	PD, CAL, BOP	1, 3 months	PD reduction CAL gain BOP reduction	SRP + EPAP were effective in a short-term period.
Jentsch, 2020, Germany [12]	RCT Parallel	*n* = 42 Control group Mean age: 54.29 ± 7.44 Female: *n* = 10 Male: *n* = 11 Test group Mean age: 50.23 ± 8.26 Female: *n* = 7 Male: *n* = 14 Diagnosis: periodontitis	Control: subgingival instrumentation Test: subgingival instrumentation + subgingival erythritol air-polishing	PD, CAL, BOP	3, 6 months	PD reduction CAL gain BOP reduction	The adjunctive use of erythritol air-polishing may add benefits in subgingival instrumentation.
Mensi, 2021, Italy [13]	RCT Parallel	*n* = 36 Control group Mean age: 48.44 ± 9.31 Female: *n* = 11 Male: *n* = 7 Test group Mean age: 52.06 ± 10.17 Female: *n* = 7 Male: *n* = 11 Diagnosis: periodontitis stage III–IV	Control: ultrasonic instrumentation + supragingival erythritol powder air-polishing Test: ultrasonic instrumentation + supragingival and subgingival erythritol powder air-polishing	PD, CAL, BOP	3 months	PD reduction CAL gain BOP reduction	The addition of subgingival erythritol powder air-polishing does not provide significant advantages.
Stein, 2021, Germany [14]	RCT Parallel	*n* = 180 Q-SRP: *n* = 35 Mean age: 57.8 ± 11.1 Female: *n* = 13 Male: *n* = 22 FMS: *n* = 47 Mean age: 53.4 ± 10.8 Female: *n* = 26 Male: *n* = 21 FMD: *n* = 43 Mean age: 51.8 ± 13.0 Female: *n* = 23 Male: *n* = 20 FMDAP: *n* = 47 Mean age: 49.9 ± 11.9 Female: *n* = 24 Male: *n* = 23 Diagnosis: periodontitis stage III–IV	Q-SRP: quadrant-wise SRP FMS: full-mouth scaling FMD: full-mouth disinfection FMDAP: FMD with adjuvant erythritol air-polishing	PPD, CAL, BOP	3, 6 months	PPD reduction CAL gain BOP reduction	All four protocols showed clinical improvements. The addition of erythritol air-polishing in FMDAP resulted in better outcomes compared to Q-SRP.
Supportive periodontal therapy							
Hägi, 2013, Switzerland [15]	RCT Parallel	*n* = 40 Control group Mean age: 53.7 ± 10.09 Female: *n* = 8 Male: *n* = 12 Test group Mean age: 55.2 ± 7.97 Female: *n* = 7 Male: *n* = 13 Diagnosis: moderate to advanced chronic periodontitis	Control: SRP Test: EPAP	PPD, CAL, BOP	1, 3 months	BOP reduction PPD reduction CAL gain	EPAP may be considered as a modality treatment for repeated instrumentation in supportive periodontal therapy.
Muller, 2014, Switzerland [16]	RCT Parallel	*n* = 50 Mean age: 58.5 Female: *n* = 29 Male: *n* = 21 Diagnosis: periodontal disease	Control: ultrasonic debridement Test: subgingival erythritol air-polishing	PD, BOP	3, 6, 9, 12 months	PD reduction BOP reduction	At 12 months, outcomes were not significant different.
Ulvik, 2021, Norway [17]	RCT Split mouth	*n* = 20 Mean age: 61 Female: *n* = 14 Male: *n* = 6 Diagnosis: moderate to severe periodontitis	Control: curette + ultrasonic treatment Test: subgingival erythritol air-polishing	PD, CAL, BOP	3, 6, 9, 12 months	PD reduction BOP reduction CAL gain	Both therapies were efficient. Control group showed superior CAL gain.

BOP, bleeding on probing; CAL, clinical attachment level; EPAP, erythritol powder air-polishing powder; NA, not available; PD, pocket depth; PPD, probing pocket depth; RCT, randomized clinical trial; SRP, scaling and root planning.

## Data Availability

Not applicable.
